# Bis{2,4-dichloro-6-[3-(dimethyl­amino)propyl­imino­meth­yl]phenolato}copper(II)

**DOI:** 10.1107/S1600536809038045

**Published:** 2009-09-26

**Authors:** Xian-Feng Huang

**Affiliations:** a School of Chemical Engineering, Jiangsu Polytechnic University, Changzhou 213164, People’s Republic of China

## Abstract

In the title complex, [Cu(C_12_H_15_Cl_2_N_2_O)_2_], the Cu^II^ ion is coordinated by one *N*,*O*-bidentate and one *N*,*N*′,*O*-tridentate Schiff base ligand, resulting in a distorted CuN_3_O_2_ square-based pyramidal coordination for the metal ion, with the O atoms lying *trans* to each other in the basal plane.

## Related literature

For background on Schiff bases, see: Shi *et al.* (2007 ▶, 2008 ▶). For reference structural data, see: Allen *et al.* (1987 ▶).
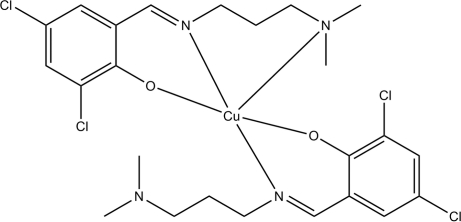


## Experimental

### 

#### Crystal data

[Cu(C_12_H_15_Cl_2_N_2_O)_2_]
*M* *_r_* = 611.86
Triclinic, 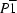

*a* = 9.4099 (17) Å
*b* = 12.548 (2) Å
*c* = 12.603 (2) Å
α = 103.271 (7)°
β = 110.907 (7)°
γ = 90.614 (8)°
*V* = 1346.0 (4) Å^3^
*Z* = 2
Mo *K*α radiation
μ = 1.24 mm^−1^
*T* = 296 K
0.28 × 0.24 × 0.15 mm

#### Data collection

Enraf–Nonius CAD-4 diffractometer
Absorption correction: ψ scan (North *et al.*, 1968 ▶) *T*_min_ = 0.723, *T*_max_ = 0.836
7433 measured reflections
5221 independent reflections
3718 reflections with *I* > 2σ(*I*)
*R*_int_ = 0.022
200 standard reflections every 3 reflections intensity decay: 1%

#### Refinement

*R*[*F* ^2^ > 2σ(*F* ^2^)] = 0.039
*wR*(*F* ^2^) = 0.112
*S* = 1.03
5221 reflections
320 parameters
H-atom parameters constrained
Δρ_max_ = 0.39 e Å^−3^
Δρ_min_ = −0.63 e Å^−3^

### 

Data collection: *CAD-4 Software* (Enraf–Nonius, 1989 ▶); cell refinement: *CAD-4 Software*; data reduction: *XCAD4* (Harms & Wocadlo, 1995 ▶); program(s) used to solve structure: *SHELXS97* (Sheldrick, 2008 ▶); program(s) used to refine structure: *SHELXL97* (Sheldrick, 2008 ▶); molecular graphics: *SHELXTL* (Sheldrick, 2008 ▶); software used to prepare material for publication: *SHELXTL*.

## Supplementary Material

Crystal structure: contains datablocks global, I. DOI: 10.1107/S1600536809038045/hb5109sup1.cif

Structure factors: contains datablocks I. DOI: 10.1107/S1600536809038045/hb5109Isup2.hkl

Additional supplementary materials:  crystallographic information; 3D view; checkCIF report

## Figures and Tables

**Table 1:** Selected bond lengths (Å)

Cu1—O2	1.921 (2)
Cu1—O1	1.924 (2)
Cu1—N1	2.003 (2)
Cu1—N3	2.009 (2)
Cu1—N2	2.459 (3)

**Fig. 1.**
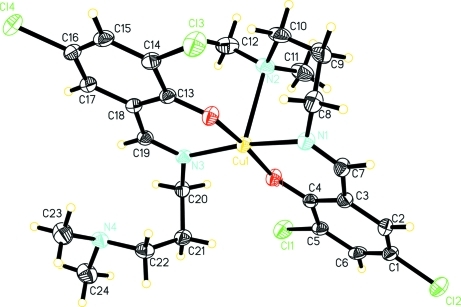
The molecular structure of (I) showing 30% probability displacement ellipsoids.

## supplementary crystallographic information

### Comment

There has been much research interest in Schiff base metal complexes due to their molecular architectures and biological activities (Shi *et al.*, 2007; Shi *et al.*, 2008). In this work, we report here the crystal structure of the title compound, (I). In (I), all bond lengths are within normal ranges (Allen *et al.*, 1987) (Fig. 1). The Cu^II^ is coordinated by two O and three N atoms from the two Schiff base ligands, forming a distorted square-pyramidal coordination (Table 1).

### Experimental

A mixture of 3,5-dichloro-2-hydroxybenzaldehyde (380 mg, 2 mmol), *N*,*N*-dimethylpropane-1,3-diamine (204 mg, 2 mmol) and CuCl_2_.4H_2_O (1 mmol, 169 mg) was stirred in methanol (10 ml) for 1 h. After keeping the filtrate in air for 8 d, green block-shaped crystals of (I) were formed.

### Refinement

All H atoms were positioned geometrically (C—H = 0.93–0.96 Å) and refined as riding, with *U*_iso_(H) = 1.2*U*_eq_(C).

### Crystal data

**Table:**

[Cu(C_12_H_15_Cl_2_N_2_O)_2_]	*Z* = 2
*M**_r_* = 611.86	*F*(000) = 630
Triclinic, *P*1	*D*_x_ = 1.510 Mg m^−^^3^
Hall symbol: -P 1	Mo *K*α radiation, λ = 0.71073 Å
*a* = 9.4099 (17) Å	Cell parameters from 25 reflections
*b* = 12.548 (2) Å	θ = 9–12°
*c* = 12.603 (2) Å	µ = 1.24 mm^−^^1^
α = 103.271 (7)°	*T* = 296 K
β = 110.907 (7)°	Block, green
γ = 90.614 (8)°	0.28 × 0.24 × 0.15 mm
*V* = 1346.0 (4) Å^3^	

### Data collection

**Table:**

Enraf–Nonius CAD-4 diffractometer	3718 reflections with *I* > 2σ(*I*)
Radiation source: fine-focus sealed tube	*R*_int_ = 0.022
graphite	θ_max_ = 26.0°, θ_min_ = 1.7°
ω/2θ scans	*h* = −11→9
Absorption correction: ψ scan (North *et al.*, 1968)	*k* = −15→15
*T*_min_ = 0.723, *T*_max_ = 0.836	*l* = −15→15
7433 measured reflections	200 standard reflections every 3 reflections
5221 independent reflections	intensity decay: 1%

### Refinement

**Table:**

Refinement on *F*^2^	Primary atom site location: structure-invariant direct methods
Least-squares matrix: full	Secondary atom site location: difference Fourier map
*R*[*F*^2^ > 2σ(*F*^2^)] = 0.039	Hydrogen site location: inferred from neighbouring sites
*wR*(*F*^2^) = 0.112	H-atom parameters constrained
*S* = 1.02	*w* = 1/[σ^2^(*F*_o_^2^) + (0.0569*P*)^2^ + 0.2468*P*] where *P* = (*F*_o_^2^ + 2*F*_c_^2^)/3
5221 reflections	(Δ/σ)_max_ < 0.001
320 parameters	Δρ_max_ = 0.39 e Å^−^^3^
0 restraints	Δρ_min_ = −0.63 e Å^−^^3^

### Special details

**Table:**

Geometry. All e.s.d.'s (except the e.s.d. in the dihedral angle between two l.s. planes) are estimated using the full covariance matrix. The cell e.s.d.'s are taken into account individually in the estimation of e.s.d.'s in distances, angles and torsion angles; correlations between e.s.d.'s in cell parameters are only used when they are defined by crystal symmetry. An approximate (isotropic) treatment of cell e.s.d.'s is used for estimating e.s.d.'s involving l.s. planes.
Refinement. Refinement of *F*^2^ against ALL reflections. The weighted *R*-factor *wR* and goodness of fit *S* are based on *F*^2^, conventional *R*-factors *R* are based on *F*, with *F* set to zero for negative *F*^2^. The threshold expression of *F*^2^ > σ(*F*^2^) is used only for calculating *R*-factors(gt) *etc*. and is not relevant to the choice of reflections for refinement. *R*-factors based on *F*^2^ are statistically about twice as large as those based on *F*, and *R*- factors based on ALL data will be even larger.

### Fractional atomic coordinates and isotropic or equivalent isotropic displacement parameters (Å^2^)

**Table:**

	*x*	*y*	*z*	*U*_iso_*/*U*_eq_	
C1	1.0980 (3)	0.3757 (3)	0.4415 (3)	0.0422 (8)	
C2	0.9891 (3)	0.2919 (3)	0.4160 (3)	0.0413 (7)	
H2	1.0083	0.2402	0.4606	0.050*	
C3	0.8480 (3)	0.2824 (2)	0.3233 (3)	0.0370 (7)	
C4	0.8149 (3)	0.3598 (2)	0.2532 (3)	0.0333 (6)	
C5	0.9332 (3)	0.4456 (2)	0.2853 (3)	0.0368 (7)	
C6	1.0705 (3)	0.4550 (3)	0.3772 (3)	0.0419 (8)	
H6	1.1440	0.5135	0.3961	0.050*	
C7	0.7331 (4)	0.1966 (3)	0.3072 (3)	0.0412 (7)	
H7	0.7569	0.1558	0.3632	0.049*	
C8	0.4966 (4)	0.0924 (3)	0.2371 (3)	0.0522 (9)	
H8A	0.5532	0.0519	0.2930	0.063*	
H8B	0.4442	0.0400	0.1623	0.063*	
C9	0.3798 (4)	0.1536 (3)	0.2794 (3)	0.0659 (11)	
H9A	0.3207	0.1011	0.2971	0.079*	
H9B	0.4353	0.2075	0.3524	0.079*	
C10	0.2686 (4)	0.2124 (3)	0.1978 (3)	0.0548 (9)	
H10A	0.2111	0.1587	0.1251	0.066*	
H10B	0.1966	0.2420	0.2332	0.066*	
C11	0.4186 (5)	0.3915 (3)	0.2752 (3)	0.0642 (11)	
H11A	0.4538	0.4524	0.2536	0.096*	
H11B	0.5045	0.3644	0.3266	0.096*	
H11C	0.3499	0.4154	0.3147	0.096*	
C12	0.2181 (4)	0.3478 (3)	0.0858 (4)	0.0632 (11)	
H12A	0.1488	0.3794	0.1217	0.095*	
H12B	0.1634	0.2898	0.0177	0.095*	
H12C	0.2630	0.4035	0.0630	0.095*	
C13	0.2720 (3)	0.0918 (2)	−0.0966 (3)	0.0333 (6)	
C14	0.1557 (3)	0.0043 (2)	−0.1296 (3)	0.0383 (7)	
C15	0.0300 (4)	−0.0163 (3)	−0.2318 (3)	0.0428 (8)	
H15	−0.0437	−0.0744	−0.2497	0.051*	
C16	0.0141 (3)	0.0509 (3)	−0.3084 (3)	0.0433 (8)	
C17	0.1206 (3)	0.1374 (3)	−0.2816 (3)	0.0404 (7)	
H17	0.1079	0.1819	−0.3333	0.048*	
C18	0.2487 (3)	0.1596 (2)	−0.1769 (3)	0.0349 (7)	
C19	0.3603 (3)	0.2499 (2)	−0.1558 (3)	0.0354 (7)	
H19	0.3448	0.2842	−0.2165	0.042*	
C20	0.5819 (3)	0.3732 (2)	−0.0704 (3)	0.0369 (7)	
H20A	0.6133	0.4327	0.0004	0.044*	
H20B	0.5278	0.4034	−0.1363	0.044*	
C21	0.7230 (3)	0.3241 (3)	−0.0862 (3)	0.0425 (8)	
H21A	0.7862	0.3808	−0.0946	0.051*	
H21B	0.7821	0.3016	−0.0159	0.051*	
C22	0.6870 (4)	0.2265 (3)	−0.1906 (3)	0.0446 (8)	
H22A	0.7819	0.2025	−0.1970	0.054*	
H22B	0.6344	0.1664	−0.1782	0.054*	
C23	0.5059 (5)	0.1530 (3)	−0.3886 (3)	0.0611 (10)	
H23A	0.4440	0.1730	−0.4588	0.092*	
H23B	0.4413	0.1185	−0.3593	0.092*	
H23C	0.5756	0.1028	−0.4057	0.092*	
C24	0.6815 (5)	0.3107 (3)	−0.3461 (3)	0.0604 (10)	
H24A	0.7537	0.2646	−0.3658	0.091*	
H24B	0.7353	0.3763	−0.2877	0.091*	
H24C	0.6145	0.3303	−0.4150	0.091*	
Cl1	0.90023 (10)	0.54424 (7)	0.20416 (9)	0.0563 (2)	
Cl2	1.27444 (10)	0.38562 (9)	0.55552 (8)	0.0654 (3)	
Cl3	0.17577 (11)	−0.08122 (7)	−0.03566 (8)	0.0597 (3)	
Cl4	−0.14445 (10)	0.02090 (9)	−0.43971 (8)	0.0639 (3)	
Cu1	0.52922 (4)	0.23541 (3)	0.08518 (3)	0.03432 (13)	
N1	0.6029 (3)	0.1709 (2)	0.2244 (2)	0.0400 (6)	
N2	0.3386 (3)	0.3034 (2)	0.1693 (2)	0.0460 (7)	
N3	0.4784 (3)	0.28772 (18)	−0.0621 (2)	0.0331 (6)	
N4	0.5920 (3)	0.2517 (2)	−0.3004 (2)	0.0414 (6)	
O1	0.6871 (2)	0.35606 (17)	0.16617 (18)	0.0401 (5)	
O2	0.3892 (2)	0.10554 (16)	−0.00087 (18)	0.0400 (5)	

### Atomic displacement parameters (Å^2^)

**Table:**

	*U*^11^	*U*^22^	*U*^33^	*U*^12^	*U*^13^	*U*^23^
C1	0.0273 (16)	0.054 (2)	0.0371 (18)	0.0020 (15)	0.0087 (13)	0.0013 (15)
C2	0.0361 (18)	0.050 (2)	0.0334 (17)	0.0031 (15)	0.0078 (14)	0.0107 (15)
C3	0.0327 (16)	0.0389 (17)	0.0369 (17)	−0.0020 (13)	0.0119 (14)	0.0066 (14)
C4	0.0299 (16)	0.0358 (16)	0.0343 (16)	0.0006 (13)	0.0136 (13)	0.0064 (13)
C5	0.0307 (16)	0.0373 (17)	0.0426 (18)	−0.0012 (13)	0.0160 (14)	0.0064 (14)
C6	0.0256 (16)	0.0432 (18)	0.051 (2)	−0.0064 (14)	0.0155 (15)	−0.0016 (15)
C7	0.0425 (19)	0.0410 (18)	0.0408 (18)	0.0001 (15)	0.0109 (15)	0.0191 (15)
C8	0.051 (2)	0.050 (2)	0.054 (2)	−0.0174 (17)	0.0076 (17)	0.0292 (17)
C9	0.064 (3)	0.083 (3)	0.057 (2)	−0.025 (2)	0.025 (2)	0.027 (2)
C10	0.048 (2)	0.057 (2)	0.068 (2)	−0.0110 (17)	0.0346 (19)	0.0102 (19)
C11	0.070 (3)	0.056 (2)	0.069 (3)	−0.011 (2)	0.043 (2)	−0.007 (2)
C12	0.051 (2)	0.065 (3)	0.084 (3)	0.014 (2)	0.034 (2)	0.024 (2)
C13	0.0286 (16)	0.0334 (16)	0.0360 (17)	0.0000 (13)	0.0120 (13)	0.0053 (13)
C14	0.0353 (17)	0.0332 (16)	0.0457 (19)	−0.0014 (13)	0.0150 (15)	0.0089 (14)
C15	0.0328 (17)	0.0397 (18)	0.052 (2)	−0.0060 (14)	0.0172 (15)	0.0025 (15)
C16	0.0276 (16)	0.054 (2)	0.0412 (19)	0.0013 (15)	0.0101 (14)	0.0022 (15)
C17	0.0324 (17)	0.0500 (19)	0.0388 (18)	0.0007 (14)	0.0128 (14)	0.0121 (15)
C18	0.0293 (16)	0.0393 (17)	0.0369 (17)	0.0005 (13)	0.0132 (13)	0.0093 (14)
C19	0.0325 (16)	0.0386 (17)	0.0381 (17)	0.0011 (13)	0.0145 (14)	0.0132 (14)
C20	0.0403 (17)	0.0333 (16)	0.0357 (17)	−0.0091 (13)	0.0118 (14)	0.0103 (13)
C21	0.0342 (17)	0.0462 (19)	0.0477 (19)	−0.0060 (14)	0.0139 (15)	0.0153 (16)
C22	0.0420 (19)	0.0457 (19)	0.053 (2)	0.0043 (15)	0.0236 (16)	0.0166 (16)
C23	0.069 (3)	0.055 (2)	0.057 (2)	−0.0106 (19)	0.026 (2)	0.0059 (19)
C24	0.071 (3)	0.058 (2)	0.063 (2)	−0.0083 (19)	0.035 (2)	0.0188 (19)
Cl1	0.0476 (5)	0.0483 (5)	0.0710 (6)	−0.0102 (4)	0.0140 (4)	0.0243 (4)
Cl2	0.0340 (5)	0.0921 (8)	0.0524 (6)	−0.0029 (5)	−0.0006 (4)	0.0117 (5)
Cl3	0.0596 (6)	0.0484 (5)	0.0653 (6)	−0.0160 (4)	0.0104 (5)	0.0245 (5)
Cl4	0.0360 (5)	0.0847 (7)	0.0519 (6)	−0.0094 (5)	−0.0006 (4)	0.0083 (5)
Cu1	0.0302 (2)	0.0355 (2)	0.0352 (2)	−0.00629 (15)	0.00768 (16)	0.01242 (16)
N1	0.0374 (15)	0.0360 (14)	0.0452 (16)	−0.0081 (11)	0.0110 (13)	0.0148 (12)
N2	0.0462 (16)	0.0438 (16)	0.0524 (17)	−0.0043 (13)	0.0258 (14)	0.0084 (13)
N3	0.0291 (13)	0.0331 (14)	0.0389 (15)	−0.0021 (11)	0.0132 (12)	0.0117 (11)
N4	0.0418 (15)	0.0427 (15)	0.0434 (16)	−0.0038 (12)	0.0200 (13)	0.0111 (12)
O1	0.0332 (12)	0.0422 (12)	0.0407 (12)	−0.0090 (9)	0.0055 (10)	0.0162 (10)
O2	0.0380 (12)	0.0374 (12)	0.0393 (12)	−0.0055 (9)	0.0054 (10)	0.0142 (10)

### Geometric parameters (Å, °)

**Table:**

C1—C2	1.359 (4)	C14—C15	1.370 (4)
C1—C6	1.393 (4)	C14—Cl3	1.737 (3)
C1—Cl2	1.745 (3)	C15—C16	1.393 (4)
C2—C3	1.403 (4)	C15—H15	0.9300
C2—H2	0.9300	C16—C17	1.365 (4)
C3—C4	1.423 (4)	C16—Cl4	1.745 (3)
C3—C7	1.445 (4)	C17—C18	1.401 (4)
C4—O1	1.298 (3)	C17—H17	0.9300
C4—C5	1.419 (4)	C18—C19	1.446 (4)
C5—C6	1.375 (4)	C19—N3	1.284 (4)
C5—Cl1	1.743 (3)	C19—H19	0.9300
C6—H6	0.9300	C20—N3	1.488 (3)
C7—N1	1.272 (4)	C20—C21	1.527 (4)
C7—H7	0.9300	C20—H20A	0.9700
C8—N1	1.470 (3)	C20—H20B	0.9700
C8—C9	1.527 (5)	C21—C22	1.510 (4)
C8—H8A	0.9700	C21—H21A	0.9700
C8—H8B	0.9700	C21—H21B	0.9700
C9—C10	1.514 (5)	C22—N4	1.461 (4)
C9—H9A	0.9700	C22—H22A	0.9700
C9—H9B	0.9700	C22—H22B	0.9700
C10—N2	1.485 (4)	C23—N4	1.459 (4)
C10—H10A	0.9700	C23—H23A	0.9600
C10—H10B	0.9700	C23—H23B	0.9600
C11—N2	1.470 (4)	C23—H23C	0.9600
C11—H11A	0.9600	C24—N4	1.456 (4)
C11—H11B	0.9600	C24—H24A	0.9600
C11—H11C	0.9600	C24—H24B	0.9600
C12—N2	1.462 (4)	C24—H24C	0.9600
C12—H12A	0.9600	Cu1—O2	1.921 (2)
C12—H12B	0.9600	Cu1—O1	1.924 (2)
C12—H12C	0.9600	Cu1—N1	2.003 (2)
C13—O2	1.285 (3)	Cu1—N3	2.009 (2)
C13—C14	1.421 (4)	Cu1—N2	2.459 (3)
C13—C18	1.427 (4)		
			
C2—C1—C6	120.5 (3)	C18—C17—H17	119.7
C2—C1—Cl2	120.5 (3)	C17—C18—C13	120.9 (3)
C6—C1—Cl2	119.0 (2)	C17—C18—C19	117.9 (3)
C1—C2—C3	120.7 (3)	C13—C18—C19	121.2 (3)
C1—C2—H2	119.6	N3—C19—C18	126.9 (3)
C3—C2—H2	119.6	N3—C19—H19	116.5
C2—C3—C4	121.1 (3)	C18—C19—H19	116.5
C2—C3—C7	117.7 (3)	N3—C20—C21	110.5 (2)
C4—C3—C7	121.0 (3)	N3—C20—H20A	109.5
O1—C4—C5	120.5 (3)	C21—C20—H20A	109.5
O1—C4—C3	124.4 (3)	N3—C20—H20B	109.5
C5—C4—C3	115.1 (3)	C21—C20—H20B	109.5
C6—C5—C4	123.4 (3)	H20A—C20—H20B	108.1
C6—C5—Cl1	118.8 (2)	C22—C21—C20	114.1 (3)
C4—C5—Cl1	117.7 (2)	C22—C21—H21A	108.7
C5—C6—C1	119.0 (3)	C20—C21—H21A	108.7
C5—C6—H6	120.5	C22—C21—H21B	108.7
C1—C6—H6	120.5	C20—C21—H21B	108.7
N1—C7—C3	126.8 (3)	H21A—C21—H21B	107.6
N1—C7—H7	116.6	N4—C22—C21	112.6 (3)
C3—C7—H7	116.6	N4—C22—H22A	109.1
N1—C8—C9	110.1 (3)	C21—C22—H22A	109.1
N1—C8—H8A	109.6	N4—C22—H22B	109.1
C9—C8—H8A	109.6	C21—C22—H22B	109.1
N1—C8—H8B	109.6	H22A—C22—H22B	107.8
C9—C8—H8B	109.6	N4—C23—H23A	109.5
H8A—C8—H8B	108.2	N4—C23—H23B	109.5
C10—C9—C8	117.6 (3)	H23A—C23—H23B	109.5
C10—C9—H9A	107.9	N4—C23—H23C	109.5
C8—C9—H9A	107.9	H23A—C23—H23C	109.5
C10—C9—H9B	107.9	H23B—C23—H23C	109.5
C8—C9—H9B	107.9	N4—C24—H24A	109.5
H9A—C9—H9B	107.2	N4—C24—H24B	109.5
N2—C10—C9	115.5 (3)	H24A—C24—H24B	109.5
N2—C10—H10A	108.4	N4—C24—H24C	109.5
C9—C10—H10A	108.4	H24A—C24—H24C	109.5
N2—C10—H10B	108.4	H24B—C24—H24C	109.5
C9—C10—H10B	108.4	O2—Cu1—O1	173.30 (9)
H10A—C10—H10B	107.5	O2—Cu1—N1	89.22 (9)
N2—C11—H11A	109.5	O1—Cu1—N1	90.08 (9)
N2—C11—H11B	109.5	O2—Cu1—N3	90.21 (9)
H11A—C11—H11B	109.5	O1—Cu1—N3	89.49 (9)
N2—C11—H11C	109.5	N1—Cu1—N3	171.46 (10)
H11A—C11—H11C	109.5	O2—Cu1—N2	87.36 (9)
H11B—C11—H11C	109.5	O1—Cu1—N2	99.17 (9)
N2—C12—H12A	109.5	N1—Cu1—N2	82.79 (10)
N2—C12—H12B	109.5	N3—Cu1—N2	105.69 (9)
H12A—C12—H12B	109.5	C7—N1—C8	116.5 (3)
N2—C12—H12C	109.5	C7—N1—Cu1	125.1 (2)
H12A—C12—H12C	109.5	C8—N1—Cu1	118.3 (2)
H12B—C12—H12C	109.5	C12—N2—C11	109.4 (3)
O2—C13—C14	120.6 (3)	C12—N2—C10	108.9 (3)
O2—C13—C18	124.1 (3)	C11—N2—C10	111.1 (3)
C14—C13—C18	115.3 (3)	C12—N2—Cu1	110.2 (2)
C15—C14—C13	123.3 (3)	C11—N2—Cu1	107.1 (2)
C15—C14—Cl3	118.8 (2)	C10—N2—Cu1	110.2 (2)
C13—C14—Cl3	117.9 (2)	C19—N3—C20	114.8 (2)
C14—C15—C16	119.1 (3)	C19—N3—Cu1	124.33 (19)
C14—C15—H15	120.4	C20—N3—Cu1	120.80 (18)
C16—C15—H15	120.4	C24—N4—C23	110.6 (3)
C17—C16—C15	120.7 (3)	C24—N4—C22	111.8 (3)
C17—C16—Cl4	121.0 (3)	C23—N4—C22	111.9 (3)
C15—C16—Cl4	118.3 (2)	C4—O1—Cu1	128.22 (18)
C16—C17—C18	120.6 (3)	C13—O2—Cu1	128.34 (18)
C16—C17—H17	119.7		
